# Synthesis of Both Ionic Species of Ammonium Dithiocarbamate Derived Cholic Acid Moieties

**DOI:** 10.3390/molecules16086306

**Published:** 2011-07-26

**Authors:** Juha Koivukorpi, Erkki Kolehmainen

**Affiliations:** Department of Chemistry, FI-40014 University of Jyväskylä, Finland

**Keywords:** cholic acid, dithiocarbamate, steroid, NMR spectroscopy, amine

## Abstract

The reaction of 3-aminopropylamide of cholic acid with CS_2_ produced a bile acid derivative of dithiocarbamic acid which further formed an ammonium salt with another molecule of 3-aminopropylamide of cholic acid. The cationic 3-ammonium propylamide of cholic acid did not react further with CS_2_ and the formed salt was stable in the reaction mixture, even when excess CS_2_ was used. When the reaction was carried out in the presence of aqueous sodium hydroxide, only the bile acid derivative of sodium dithiocarbamate was formed. The dithiocarbamate derivatives were characterized by ^1^H- and ^13^C-NMR spectroscopy and ESI-TOF mass spectrometry.

## 1. Introduction

**Scheme 1:**
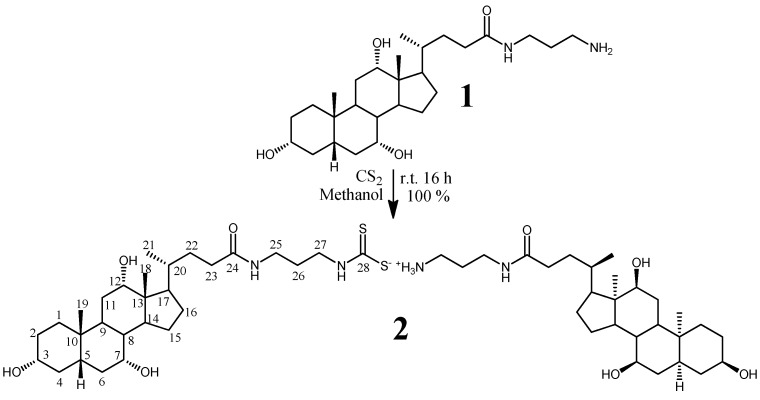
Synthesis and numbering of **2**.

It is known that amines react with CS_2_ forming dithiocarbamates [1,2,3,4,5,6]. Recently, Yavari *et al.* [7] have reported new synthetic strategies to prepare dithiocarbamates due to their large application potential. Dithiocarbamates exhibit numerous biological activities [9,10,11,12] and they are used in agriculture [12,13,14] and as linkers in solid-phase organic synthesis [15,16,17]. Further, dithiocarbamates are also widely used in medicinal chemistry and they have found application in cancer therapy [18,19]. As a biologically important expansion of the topic, we now present the synthesis and characterization of cholic acid-derived ammonium dithiocarbamates. This straightforward synthetic method offers an effective route to bile acid derivatives with increased water solubility, which is an essential property in the design of physiologically active receptors [20,21] and drug carriers [22]. The synthetic route and numbering of **2** are shown in Scheme 1.

## 2. Results and Discussion

### 2.1. Chemistry

As the first step, *N*-(3-aminopropyl)-3-alpha,7-alpha,12-alpha-trihydroxy-5-beta-cholan-24-amide (**1**) was prepared by a reaction of methyl cholate (methyl 3-alpha,7-alpha,12-alpha-trihydroxy-5-beta-cholan-24-oate) with 1,3-diaminopropane (7 days at r.t.) [23]. Then compound **1** was allowed to react with CS_2_ in methanol to form the desired product, *N*-(3-aminopropyl)-3-α,7-α,12-α-trihydroxy-5-β-cholan-24-oyl ditihiocarbamate of *N*-(3-ammoniumpropyl)-3-α,7-α,12-α-trihydroxy-5-β-cholan-24-oic acid amide (**2**).

### 2.2. Spectroscopy

**Figure 2:**
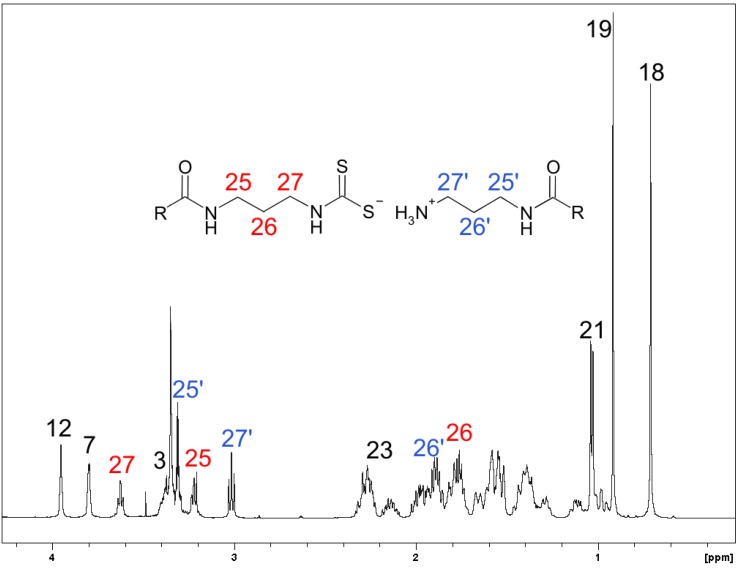
^1^H-NMR spectrum of **2** in CD_3_OD at 303 K and its partial assignment.

**Figure 3:**
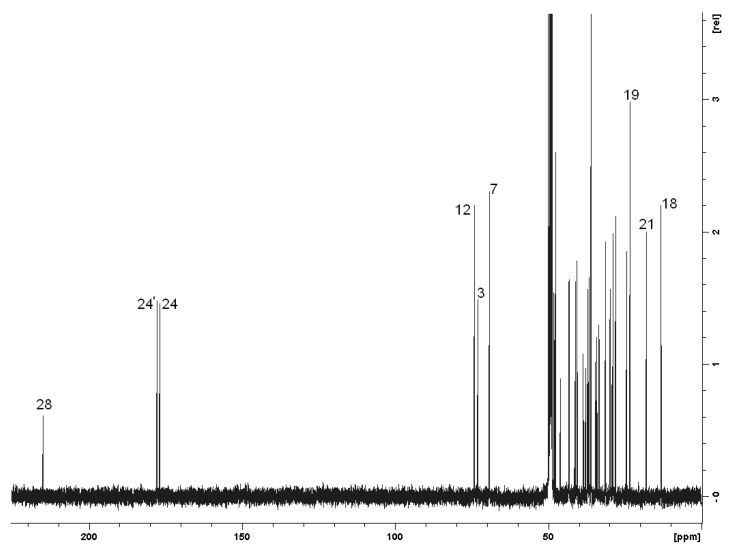
^13^C-NMR spectrum of **2** in CD_3_OD at 303 K.

The structural characterization of **2** is based on one-dimensional ^1^H-, ^13^C-, and ^13^C DEPT-135 data, as well as two-dimensional PFG DQF ^1^H-^1^H COSY [24,25,^26^], PFG ^1^H-^13^C HMQC [27,28], PFG ^1^H-^13^C HMBC [29] NMR studies and mass spectra, as well as elemental analysis. The ^1^H- and ^13^C-NMR spectra of the sodium dithiocarbamate derivative of cholic acid 3-aminopropylamide was also used to distinguish the assignments of anionic and cationic parts.^1^H-NMR spectral assignments of non-steroidal parts and some of the steroidal part are presented in Figure 2, where the signals marked in blue coming from the cationic part and those marked in red from the anionic part, respectively. Their unambiguous assignments are based on the comparison of the ^1^H-NMR spectra of **1** with the cholic acid-derived sodium dithiocarbamate prepared in the presence of aqueous sodium hydroxide and PFG DQF ^1^H,^1^H COSY correlations. The signals originating from protons 3, 7, 12, Me-18, Me-19, Me-21 and 23 of the bile acid moieties in both ionic species overlap. For the assignment of the ^13^C-NMR spectrum (Figure 3 and Table 1) the reference data [30] and heteronuclear chemical shift correlation measurements PFG ^1^H,^13^C HMQC and HMBC were used. Unfortunately the ammonium dithiocarbamate moiety of **2** was not thermally stable and it degraded in one week, whereas the sodium salt remained unchanged for several months when stored at room temperature.

**Table 1:** ^13^C NMR chemical shifts (±0.1 ppm) of **2** from int. TMS in CD_3_OD at 303 K.

Carbon	δ (ppm)	Carbon	δ (ppm)
1,1’	36.6	15,15’	24.4
2,2’	31.3	16,16’	28.8
3,3’	73.0	17,17’	48.2,48.1
4,4’	40.6	18,18’	13.2
5,5’	43.3	19,19’	23.4
6,6’	37.0	20,20’	37.2
7,7’	69.1	21,21’	18.0
8,8’	41.1	22,22’	33.5,33.4
9,9’	28.0	23,23’	34.5,34.2
10,10’	36.0	24,24’	176.8,177.6
11,11’	29.9	25,25’	37.8,37.2
12,12’	74.0	26,26’	29.9,29.1
13,13’	47.6	27,27’	45.9,38.6
14,14’	43.1	28	214.7

## 3. Experimental

### 3.1. General

All reagents and solvents of analytical grade were purchased from Sigma-Aldrich and were used without further purification. ^1^H- and ^13^C-NMR experiments were run on a Bruker Avance DRX 500 FT NMR spectrometer equipped with a 5 mm diameter inverse detection probehead and z-gradient accessory. ^1^H chemical shifts were referenced to the center peak of the CD_2_HOD quintet δ(^1^H) = 3.31 ppm and ^13^C chemical shifts to the center peak of the CD_3_OD heptet δ(^13^C) = 49.15 ppm from the internal TMS. Mass spectra were run with a QSTAR Elite MS/MS mass spectrometer system. Elemental analyses were performed on a Perkin Elmer 2400, series II, CHNS/O analyzer.

### 3.2. Synthesis of N*-(3-aminopropyl)-3-α,7-α,12-α-trihydroxy-5-β-cholan-24-oyl ditihiocarbamate of* N*-(3-ammoniumpropyl)-3-α,7-α,12-α-trihydroxy-5-β-cholan-24-oic acid amide (**2**)*

3-Aminopropylamide of cholic acid (**1**) (100 mg, 0.22 mmol) and CS_2_ (30 μL, 0.50 mmol) were dissolved in methanol (10 mL) and stirred overnight at room temperature. After that the solvent and an excess CS_2_ was removed *in vacuo*. Yield 100%. ^1^H-NMR (methanol-d_4_): δ (ppm) = 0.71 (Me-18, 6H, s), 0.92 (Me-19, 6H, s), 1.06 (Me-21, 6H, d), 1.07-2.06 (44H), 2.08-2.20 (2H), 2.21-2.33 (6H), 3.01 (2H, t), 3,22 (2H, t), 3.31 (2H, t), 3.37 (2H, m), 3.63 (2H, t), 3.80 (2H, d), 3.95 (2H, s), 4.83 (12H). MS (ESI-TOF): m/z = 1006 [M+H]^+^, 1028 [M+Na]^+^, 465 [M-C_28_H_47_N_2_O_4_S_2_]^+^, 539 [M-C_27_H_49_N_2_O_4_]^−^. M.W. (C_55_H_96_N_4_O_8_S_2_) = 1005.50. Elemental analysis: calcd (%) for C_55_H_96_N_4_O_8_S_2_ 5 H_2_O: C, 60.30; H, 9.75; N, 5.11. Found C, 60.38; H, 9.42; N, 5.02. 

## 4. Conclusions

We have demonstrated the straightforward synthesis of the cholic acid derived ammonium dithiocarbamate in both ionic species in 100% yield. We are planning to extend this approach starting from other ω-aminoalkylamides of other bile acids. The sodium salts of their dithiocarbamates are very promising starting materials in the preparation of dithiocarbamate gold(III) complexes, which have been shown to possess cytotoxic properties. They are being evaluated as potential antitumor agents as an alternative to cisplatin [18,31,32,33].
